# Characteristics of Energy Conversion and Temperature Response of Coal Excited by a DC Electric Field

**DOI:** 10.1021/acsomega.2c03582

**Published:** 2022-10-27

**Authors:** Zhihui Wen, Libo Zhang, Jianwei Wang, Xiangyu Xu, Yunpeng Yang, Yanxia Zhao

**Affiliations:** † State Key Laboratory Cultivation Base for Gas Geology and Gas Control (Henan Polytechnic University), Jiaozuo, 454000, China; ‡ State Collaborative Innovation Center of Coal Work Safety and Clean-efficiency Utilization, Jiaozuo, 454000, China; § Wuhan University of Technology, Wuhan, Hubei430070, China; ∥ Zhengzhou Coal Industry (Group) Company Limited, Zhengzhou, 450000, China; @ School of Mathematics and Information Science, Henan Polytechnic University, Kaifeng, Henan475004, China

## Abstract

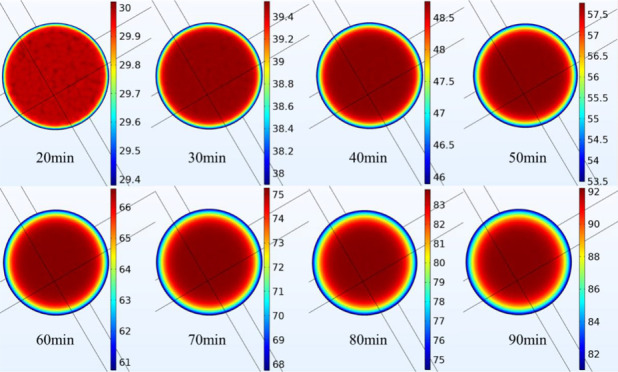

This study is aimed at investigating the characteristics of energy conversion and temperature response of coal excited by a direct current (DC) electric field (CEEDCEF). First, factors influencing energy conversion and temperature response of CEEDCEF were theoretically analyzed. Based on the analysis, the temperature distribution law of coal under different excitation conditions was simulated using the COMSOL software. The results of theoretical analysis and numerical simulation were verified through an experiment on the temperature distribution on the surface of CEEDCEF. Finally, the energy conversion mechanism and temperature response characteristics of CEEDCEF were revealed. The research results show that loading time and the loading voltage are the main factors influencing the temperature rise of CEEDCEF. Under the excitation of 6000 V constant DC voltage, the internal temperature at the lower end face of CEEDCEF increased from 29.4 to 92 °C within 20–90 min, the sections of the internal temperature increased from 36 to 94 °C under different voltage excitations of 3000–6000 V. Moreover, the temperature rise response process is divided into three stages, i.e., slow warming, fast warming, and slow cooling into stabilization. The coal shows a “capacitance effect” in the early stage of DC electric field excitation and a “resistance effect” after the charge reaches saturation. In addition, the temperature surges when the free radicals in the macromolecular structure of the coal turn into a current beam. With the increase in excitation time, the electrical parameters of the coal tend to be stable, and the surface temperature slowly decreases and stabilizes accordingly. The research results provide theoretical support for the gas production mechanism of the coal stimulated by the electric field and exploring methods for the monitoring and prewarning of these dynamic disasters.

## 1 Introduction

Coal, a medium with a dual pore structure, is a combination of natural fractures and matrix pores.^1^ In an original coal seam, gas is mostly adsorbed in coal pores, while a small portion of gas diffuses into coal fractures.^2^ As mining activities in coal seams deepen, the complexity of geological conditions, gas content, gas pressure, ground stress, and ground temperature increase, which greatly raises the possibility of coal and gas outbursts.^3,4^ Gas extraction in coal seams is an effective means to prevent gas outbursts. However, the poor permeability and low infiltration rate of coal seams greatly affect the effect of gas extraction. Therefore, the key to achieving efficient gas extraction is to inhibit gas adsorption in the coal, facilitate rapid gas desorption, and improve the permeability and infiltration rate of coal seams.^5−7^

Scholars at home and abroad have carried out extensive relevant research on the technical problems of gas extraction in deep and low-infiltration coal seams. The methods for mechanically enhancing the infiltration rate of coal seams mainly include high-pressure hydration, blast fracturing, and gas-injection displacement.^8−10^ Besides this, the methods for modifying the adsorption properties of coal by external physical fields are mainly focused on the external alternating electromagnetic field^11,12^ and the alternating electric field.^13−15^ In recent years, new research progress has been made in the fields of modifying the gas adsorption properties of coal by the external electric field and enhancing the infiltration rate by electrically fracturing the coal.

The adsorption potential energy between the coal surface and the methane molecules is reduced by the displacement polarization of electrons and ions in the coal under the excitation of an external electric field and by the excitation of electrons and ions; as a result, the adsorption capacity of coal for methane is changed.^16,17^ He et al.^18^ studied the effect of the alternating electromagnetic field on gas adsorption characteristics. The results showed that when the electric field strength reaches 20 kV/m and the frequency reaches 6–8 MHz, gas desorption can be accelerated, which increases the initial gas desorption amount by 40%–70%; meanwhile, this electromagnetic field can enhance the permeability of coal seams and reduce the gas adsorption amount. Yi et al.^19^ found that, under the action of an external alternating electric field, the value of saturation adsorption constant *a* does not vary with the external voltage; besides this, the adsorption potential trap on the coal surface becomes shallower, which leads to a decrease in the amount of gas adsorbed by the coal. Lei et al.^20^ performed an adsorption–desorption experiment on the coal rock under the condition of high-intensity electricity. The results revealed that pores and fractures in the coal rock develop and the gas desorption amount increases under the action of a high-intensity electric field. From the perspective of energy, after the external electric field acts on the coal, on the one hand, the electric field energy induces its internal dielectric consumption. On the other hand, the Joule heat effect raises its temperature significantly, leading to a competition between adsorption potential traps on the coal surface. The above two factors jointly enhance the desorption and diffusion capacities for gas. Ultimately, the electrical conductivity of the coal is augmented.^21,22^ Yang^23^ discussed the relationship between the temperature rise effect and the resistivity of the anthracite surface with an external DC electric field and reached the conclusion that the temperature rise effect lags behind the change of the current that passes through the coal sample. He et al.^24^ discovered that the depletion of the dielectric in the coal caused by the alternating electromagnetic field will raise the temperature of the coal, which enhances the desorption and diffusion capacities for gas.

When the external electric field strength is higher than the breakdown field strength of the coal, the coal loses its insulating properties due to the accumulation of a sufficient number and energy of charged particles,^25^ and thus shows a “breakdown effect”. By using a test system of electro-heat-induced coal fracturing under high-voltage breakdown, Yan and Lin^26,27^ conducted a breakdown test on the coal. The experimental results revealed that the coal fractured by electro heat forms many new pores and fractures in its interior and presents a violent “sound and light” phenomenon in the breakdown process accompanied by burnt-smell gas. With the aid of a high-voltage electric pulse experimental system, Wang^28^ investigated the effect of conductive ions on the electric-pulse-induced evolution of the pore structure. They discovered that the coal samples experienced the following changes after being treated with conductive ions and electric pulse: the medium ion channels in them are fully developed, the porosity and average pore size are increased, and the coal pore structure is significantly improved. Wang and Zhao^29,30^ concluded that, under the same pressure gradient, the seepage velocity of gas under the action of an external electric field is notably higher than that in the absence of an electric field.

At present, the research on coal excitation by the external electric field are mainly focused on its physical parameters and adsorption/desorption characteristics, as well as fracturing and permeability enhancement, but this research rarely involves the investigation into the energy conversion mechanism of coal and the temperature response characteristics on coal surface. In this study, factors influencing the energy conversion and temperature rise of coal excited by the external DC electric field (CEEDCEF) were theoretically analyzed first. On this basis, a temperature rise model in an open state was constructed, and the temperature distribution law under different excitation conditions was simulated using the COMSOL software. Furthermore, the results of theoretical analysis and numerical simulation were verified through an experiment on the temperature distribution characteristics on the surface of CEEDCEF. Finally, the energy conversion mechanism and temperature response characteristics of CEEDCEF were revealed. The related research results, which can provide a theoretical basis for the permeability enhancement of low-permeability coal seams excited by the electric field, are of guiding significance for gas extraction in deep mines and for the prevention and control of coal and gas outbursts.

## 2 Theory and Models

An electric field is fundamentally characterized by the effects of force on a stationary charge, that is, it does work on a moving charge, which indicates that the electric field has energy.^31^ According to the law of energy conservation and transformation, the energy of the electric field is generated by the external transformation in the process of charged system formation. Hence, in this study, a theoretical analysis was conducted on the factors influencing the energy conversion and temperature rise of CEEDCEF.

### 2.1 Analysis on Factors Influencing the Energy Conversion and Temperature Rise of CEEDCEF

**Figure 1:**
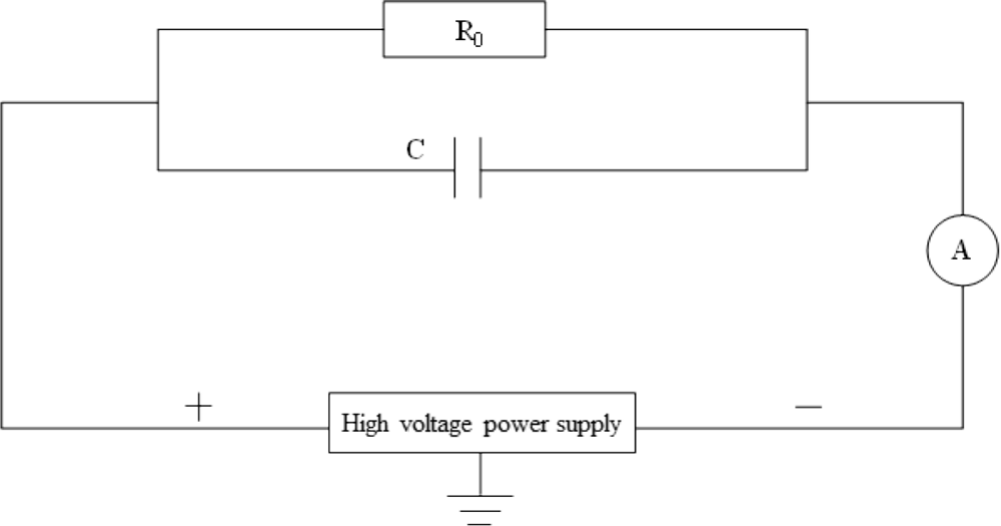
Equivalent circuit diagram of the coal.

Coal is a special dielectric material that can be considered as a parallel connection between a capacitor and a resistor when it is loaded with a DC electric field (Figure 1). At the beginning of the action of the external DC electric field, the coal mainly presents a “capacitance effect”. After being charged under the action of a high-voltage DC electric field, the coal mainly presents a “resistance effect”. The discussion on the energy conversion and the temperature change of the coal under the action of the DC electric field for a long time is mainly focused on the “resistance effect”.

During coal excitation by an external DC electric field, the total energy input from the electric field into the coal can be expressed as
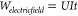
1where *W*_*electric field*_ is the energy input into the coal by the electric field, J; *U* is the voltage loaded at both ends of the coal, V; *I* is the current through the coal, A; *t* is the loading time of the electric field, s.

Since the equivalent resistance of the coal changes dynamically during the excitation, the law of Joule heat cannot be used directly in this state. Therefore, calculus is used to solve for the cumulative heat production of the coal during the excitation. As the voltage *U* loaded at both ends of the coal is always constant, the change of the current *I* at each moment can be read by the ammeter in Figure 1. Since *I* = *U*/*R* (*R* is the resistivity of the coal, Ω), it is possible to establish 1/*R* as a function of time *t*. The functional relationship between resistance *R* and time *t* can be expressed as
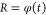
2The heat produced during the excitation can be expressed as
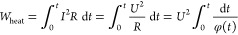
3The heat absorbed by the coal can be expressed as
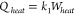
4where *k*_1_ is the conversion efficiency between the heats generated and absorbed by the coal, %.

The variation of the temperature of CEEDCEF can be expressed as
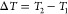
5where *ΔT* is the variation of temperature, °C; *T*_2_ is the temperature after warming, °C; and *T*_1_ is the initial temperature, °C.

Equation 6 can be obtained from the equivalent specific heat capacity of the coal.
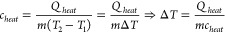
6where *c*_*heat*_ is the equivalent specific heat capacity of the coal, J/(kg·°C); *Q*_*heat*_ is the heat absorbed by CEEDCEF, J; and *m* is the mass of the coal, kg.

By combining eqs 3, 4, and 6, the relationship between the temperature rise of the coal and the voltage of the external electric field is obtained as
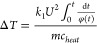
7From eq 7, it can be seen that, under the condition of a certain conversion efficiency between the heats generated and absorbed by the coal, the voltage of the excitation and the time of the action are the two key factors of the temperature rise of CEEDCEF.

### 2.2 Geometry Model and Meshing

In recent years, COMSOL Multiphysics has been well applied to the mining industry because of its unique advantages and powerful simulation functions. In this paper, this software was used to investigate the law of temperature distribution of the coal under different excitation conditions (i.e., voltage 3–6 kV, time 20–90 min). To simplify the model of the temperature response of CEEDCEF and to reduce the difficulty and time required for simulation, the following reasonable simplifications and assumptions were made.

(1) The environment where the coal samples are located is open, exchanging heat with its surrounding environment.
(2) The coal is a continuous, homogeneous, and isotropic medium.
(3) The temperature and pressure of the air domain remain constant during the heating process.
(4) The physical structure and thermophysical properties of the coal samples do not change during the heating process.

**Figure 2:**
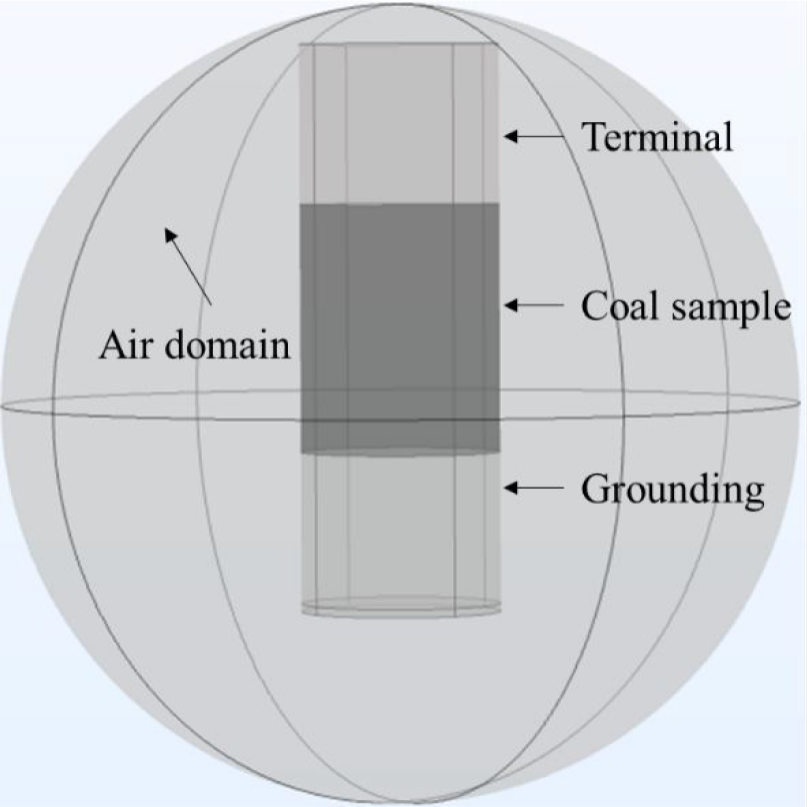
Geometric model of simulation.

As shown in Figure 2, the constructed geometric model is an air domain sphere whose radius is 200 mm. It consists of a coal sample, a terminal, and a grounding. In detail, the upper end of the coal sample is loaded with the terminal (positive) and the lower end is connected with the grounding (negative), and the two terminals are cylindrical with radiuses of 50 mm and heights of 60 mm. The coal sample, with a radius of 50 mm and a height of 100 mm, is placed at the middle position of the model.

**Figure 3:**
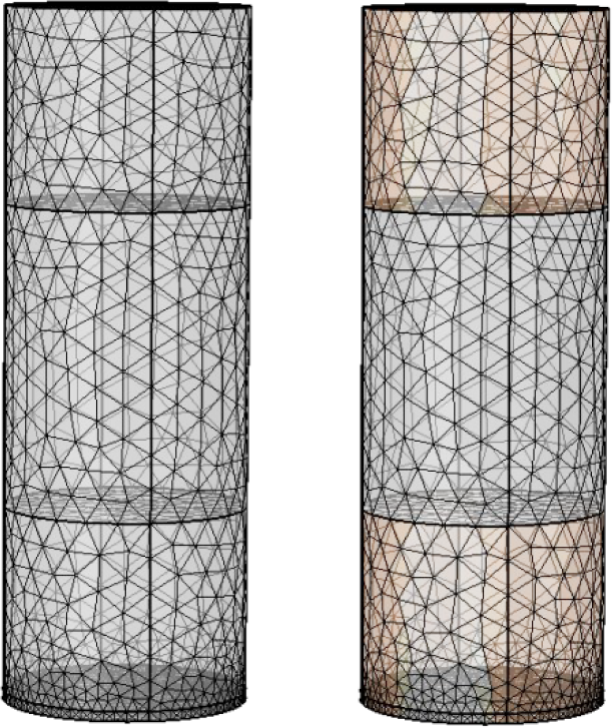
Schematic diagram of geometric model meshing.

Based on the finite element idea, discretization is performed when solving the equations. The more accurate the mesh, the higher the degree of discretization, and the smaller the deviation of the simulation results from the exact solution. The mesh quality refers to the rationality of the mesh shape, and its value ranges from 0 to 1, with 1 representing the best performance and 0 representing the worst. A total of 18,537 hyperfine-sized meshes are generated in the simulation, with an average mesh quality of 0.71. The mesh quality of the coal sample can reach over 0.88, which meets the requirements of the simulation. The distribution of the meshes divided is presented in Figure 3.

### 2.3 Governing Equation

During the action of the external DC electric field on the coal, some of the electric field energy will interconvert with the heat energy. Therefore, this process involves the energy exchange between the electric field and the temperature field of the coal sample. To simulate the heating process of the heat energy inside the coal, coupled simulation solutions are performed using partial differential equations. The governing equations involved in modeling the dual physical field of electromagnetic solid heat transfer are mainly the charge conservation equation and the temperature field equation.

Based on the charge conservation equation, the governing equation of the electric field is
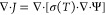
8where *J* is the current density, A/m^2^; Ψ is the electric potential, V; and *σ*(*T*) is the conductivity under temperature change, S/m.

In the calculation, the interference of the electric field on the magnetic field variation is neglected, and the variation range of the electric field strength is directly determined by the electric potential:
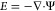
9where *E* is the electric field strength, V/m.

The governing equation for the temperature field of the coupled electric field strength is expressed as
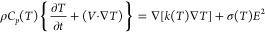
10where *E* is the electric field strength, V/m; *C_p_* is the atmospheric heat capacity, J/K; *k* is the thermal conductivity, W/(m·K); and *σ* is the electrical conductivity, S/m.

### 2.4 Boundary Conditions

Boundary conditions should be set for each physical field in the definition process for the solution. The electric field boundaries include the terminal and the grounding, with the electric potential being set to 3, 4, 5, and 6 kV. The temperature field boundaries include the electric field inlet, where the initial temperature *T*_0_ is set to 298.15 K, and the electric field outlet, which is set to a convective flow. It is assumed that the air domain medium inside the sphere will not be heated, but will exchange heat with the coal and radiate heat to an infinite distance. Detailed parameters and values are listed in Table 1.

**Table 1:** Model Parameters and Values

parameter	value	unit
conductivity of coal	0.011	S/m
relative permittivity of coal	3	1
density of coal	1350	kg/m^3^
constant pressure heat capacity of coal	4080	J/(kg·K)
coefficient of thermal expansion of coal	4.5e–5	1/K
thermal conductivity of coal	0.48	W/(m·K)
relative dielectric constant of air	1	1
conductivity of air	0	S/m
conductivity of electrode	5.998e7	S/m
thermal conductivity of electrode	400	W/(m·K)
reference temperature	293.15	K

## 3 Simulation Results

### 3.1 Different Loading Times

Due to the reasonable simplification and assumption of the established model, the temperature change inside the coal exhibits a symmetric distribution, and its upper and lower end faces show a consistent distribution. Considering the limited space, the nephograms of internal temperature at the lower end face of the coal are used here to elucidate the temperature change law of CEEDCEF at different times.

**Figure 4:**
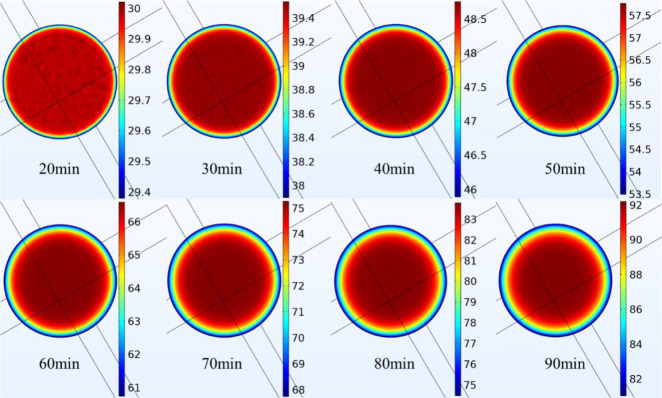
Internal temperature at the lower end face of the coal.

The nephograms of internal temperature distribution at the lower end face of CEEDCEF at 6 kV within 20–90 min are shown in Figure 4 where all the units of the temperature are degree centigrade (°C). It can be seen that the temperature ranges from 29.4 to 92 °C within 20–90 min and presents a regular distribution; specifically, the temperatures at positions closer to the center of the coal are higher. As the time of the excitation lengthens, the temperature shows an increasing trend. At 90 min, the maximum internal temperature at the lower end face can reach 90 °C.

### 3.2 Different Loading Voltages

**Figure 5:**
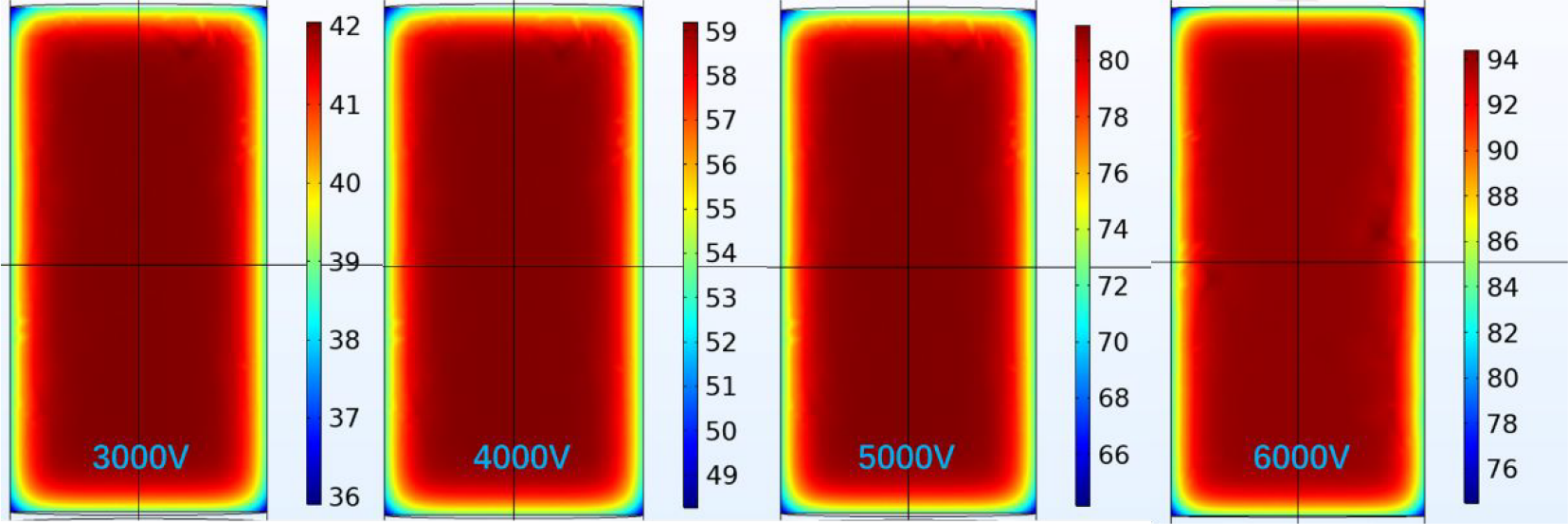
*y*–*z* temperature sections inside the coal.

The sections of the internal temperature when the 3–6 kV DC electric fields are applied to the coal for 90 min are displayed in Figure 5, and all the units of the temperature are degrees centigrade (°C). From Figure 5, the internal temperature of the coal ranges from 36 to 94 °C under the excitation voltage of 3–6 kV. Because of the heat exchange between the coal and the air domain, the temperature falls from the inside to the end face direction. By comparing the internal temperature sections at different voltages, it is found that the internal temperature of the coal gradually rises with the increase in voltage.

### 3.3 Internal Temperature Section of Coal

**Figure 6:**
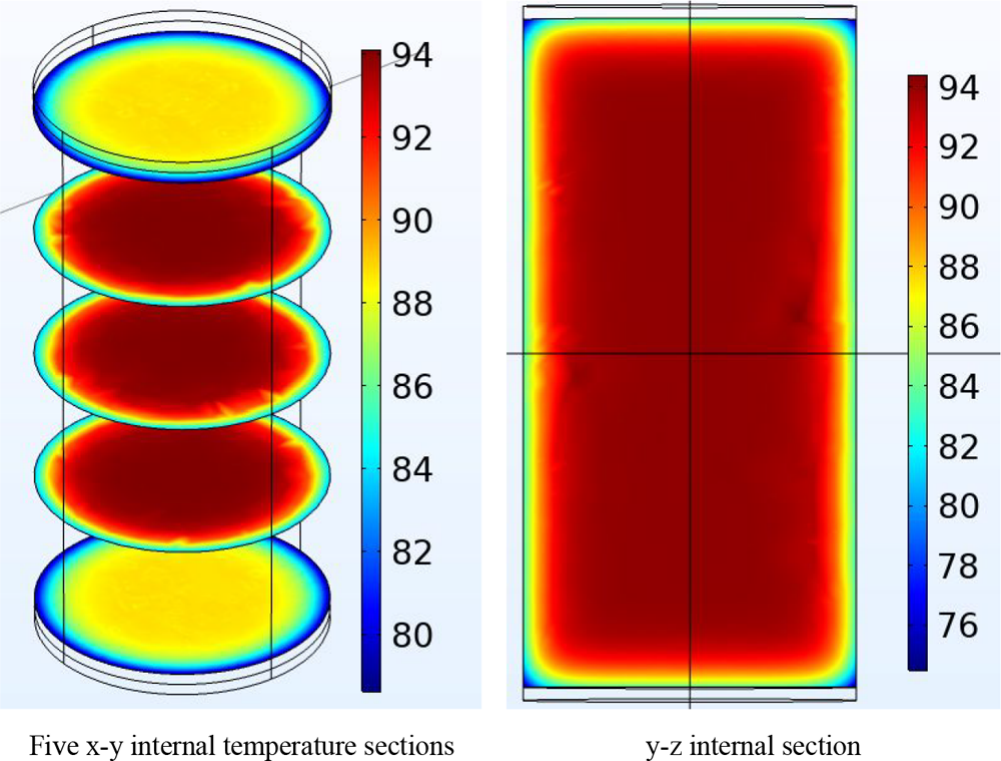
Internal temperature sections of the coal.

The temperature sections in the *x*–*y* and *y*–*z* directions inside the coal when a 6 kV DC electric field acts on it for 90 min are shown in Figure 6. At this time, the internal temperature reaches its maximum. According to Figure 6a, the maximum internal temperature of the coal reaches 94 °C in the *x*–*y* direction at 90 min. According to Figure 6b, it reaches 94 °C in the *y*–*z* direction.

## 4 Experiment on the Temperature Distribution on the Surface of CEEDCEF

For a more accurate characterization of the temperature response of CEEDCEF, an experiment was conducted to determine the temperature distribution on the surface of CEEDCEF. The principle of the temperature measurement of the infrared thermal imager is as follows: The infrared radiation signal from the target under test was received by an infrared detector, then scanned and converted into an electrical one, and finally amplified and displayed on a monitor.

**Figure 7:**
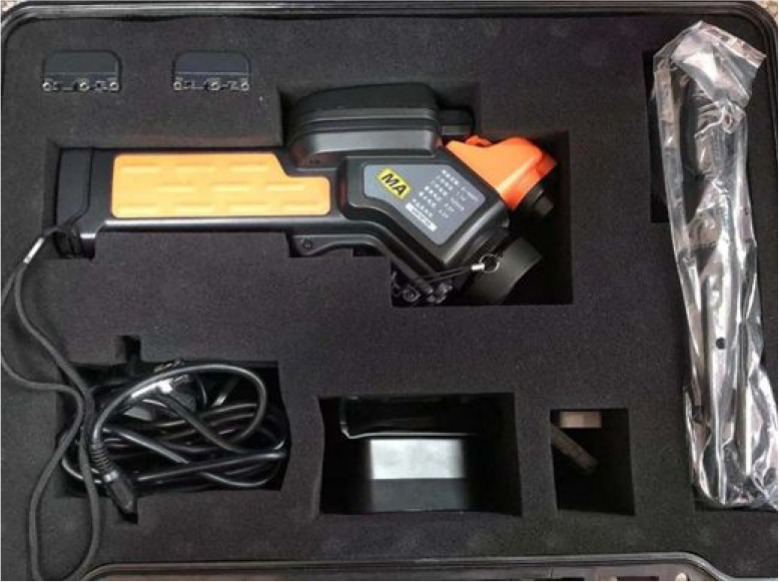
YRH600 infrared thermal imager.

Since the gripper used in the experiment was a metal cylinder that would affect the determination of temperature on the surface of the coal sample, it was unwrapped to create an open environment for the coal sample. The YRH600 infrared thermal imager (Figure 7) was used for the experiment.

### 4.1 Experimental Program

In the experiment, three types of coals were chosen as the research objects, i.e., low-rank lignite (DS) from Dongsheng Coal Mine in Ordos, Inner Mongolia, China, medium-rank long-flame coal (YM) from Yima Coal Mine, Henan Province, China, and high-rank anthracite (JZ) from Jiaozuo Coal Mine, Henan Province, China. The test results of the relevant basic parameters of the three coal samples are presented in Table 2.

**Table 2:** Basic Parameters of Experimental Coal Samples

coal sample	*R*_0_,_max_/%	FC_d_/%	*M*_ad_/%	*A*_ad_/%	*V*_daf_/%
JZ	3.34	71.64	2.30	18.74	7.32
YM	1.02	58.03	1.96	11.38	28.63
DS	0.41	41.33	7.83	14.92	35.92

**Figure 8:**
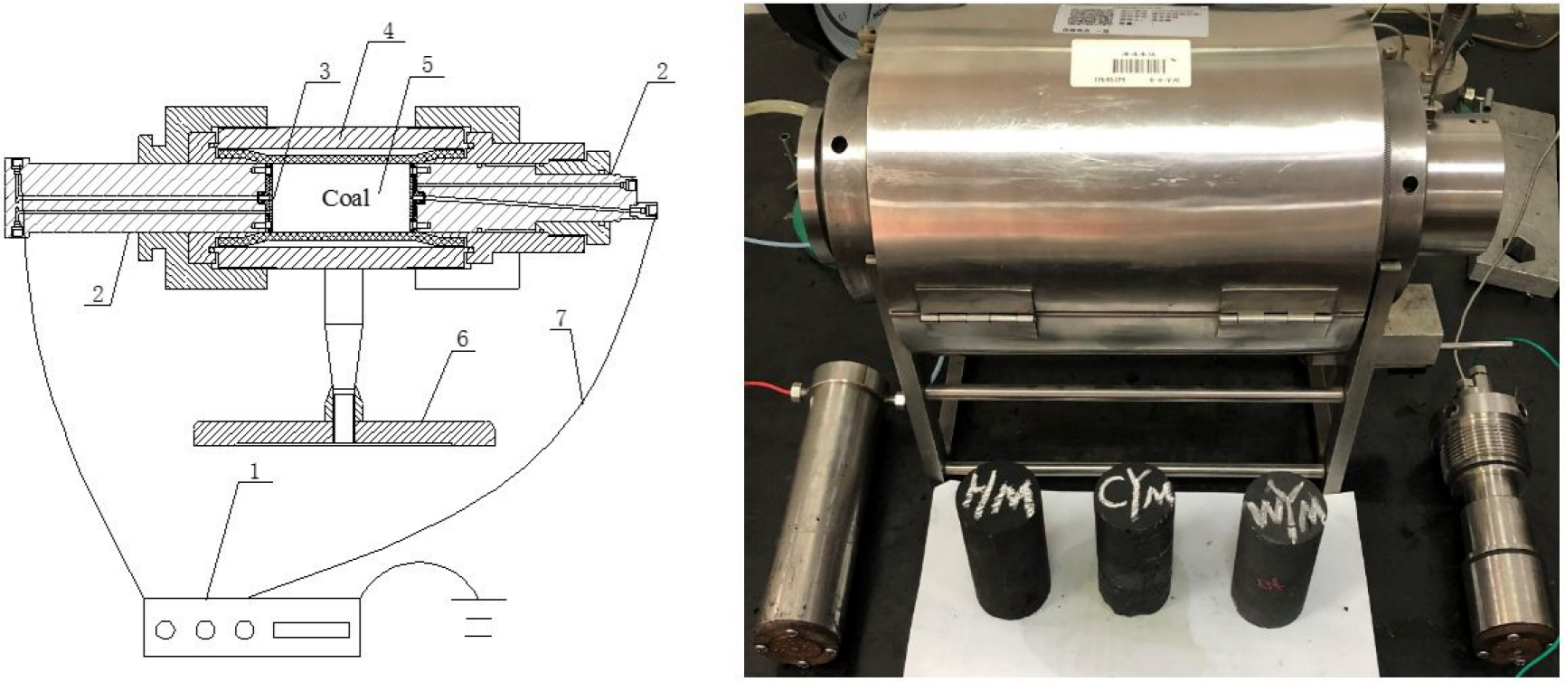
Structural diagram and photo of the test device for coal loading in the presence of an external DC electric field.

The test system of coal loading in the presence of an external DC electric field is mainly composed of a high-voltage power supply, a coal sample gripper, a rod electrode and a purple copper electrode sheet. Its structure is shown in Figure 8. The experimental procedure, parameter selection, and methods are described as follows:

(1) Installing the coal sample: The coal sample was slowly pushed into the cylinder from the side of the gripper, after which the electrodes on both sides were fixed and installed. Subsequently, an axial pressure of 0.3 MPa and a confining pressure of 0.5 MPa were loaded to make the electrodes closely fit the two end faces of the coal sample.
(2) Setting the voltage and loading time: In this experiment, the output voltage was set to 6 kV and the loading time of the electric field was set to 90 min.
(3) Debugging the infrared thermal imager: The parameters of the infrared thermal imager were as follows: temperature measurement distance 1 m, radiance 0.95, environmental temperature 7.0 °C, relative humidity 80%, and temperature correction 0.0 °C.
(4) Testing the temperature on the coal surface: The temperature on the surface of CEEDCEF was tested under the condition that the coal sample was in an open environment with the gripper unwrapped. Meanwhile, the surface of the coal was photographed every 5 min using the infrared thermal imager and measured continuously for more than 90 min.
(5) Processing the coal sample: After the experiment, the sample was removed, placed in a sealed bag, and labeled.

### 4.2 Experimental Results

According to the experimental results, the temperature rise on the surface of the coal sample is a dynamic change process, and the temperature distribution is found to be heterogeneous according to the nephograms obtained by the infrared thermal imager. As a result, the obtained infrared nephograms were processed by the SatIrWizard software and comparatively analyzed to draw the temperature rise curves of coal samples with different metamorphic degrees.

**Figure 9:**
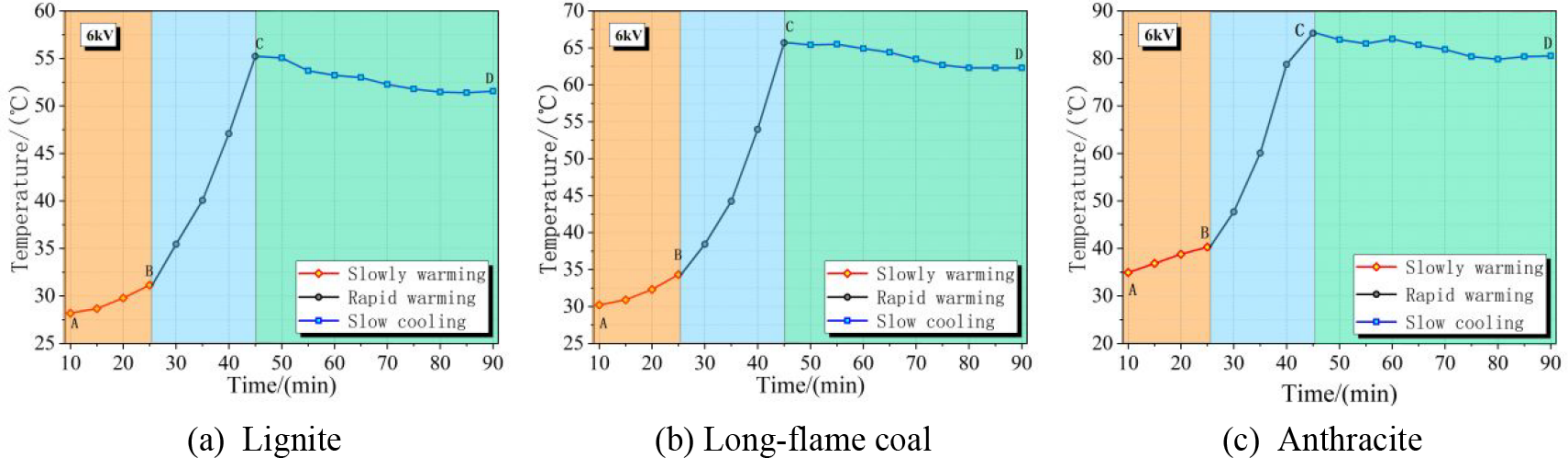
Temperature rise curves of coal samples with lignite, long-flame coal, and anthracite under a voltage of 6 kV.

The surface temperature change curves of lignite, long-flame coal, and anthracite within 90 min under the action of a 6 kV DC electric field are shown in Figure 9. It can be seen that the surface temperature rises in three stages, i.e., slow warming (10–25 min, A–B section), rapid warming (25–45 min, B–C), and slow cooling into stabilization (45–90 min, C–D).

**Figure 10:**
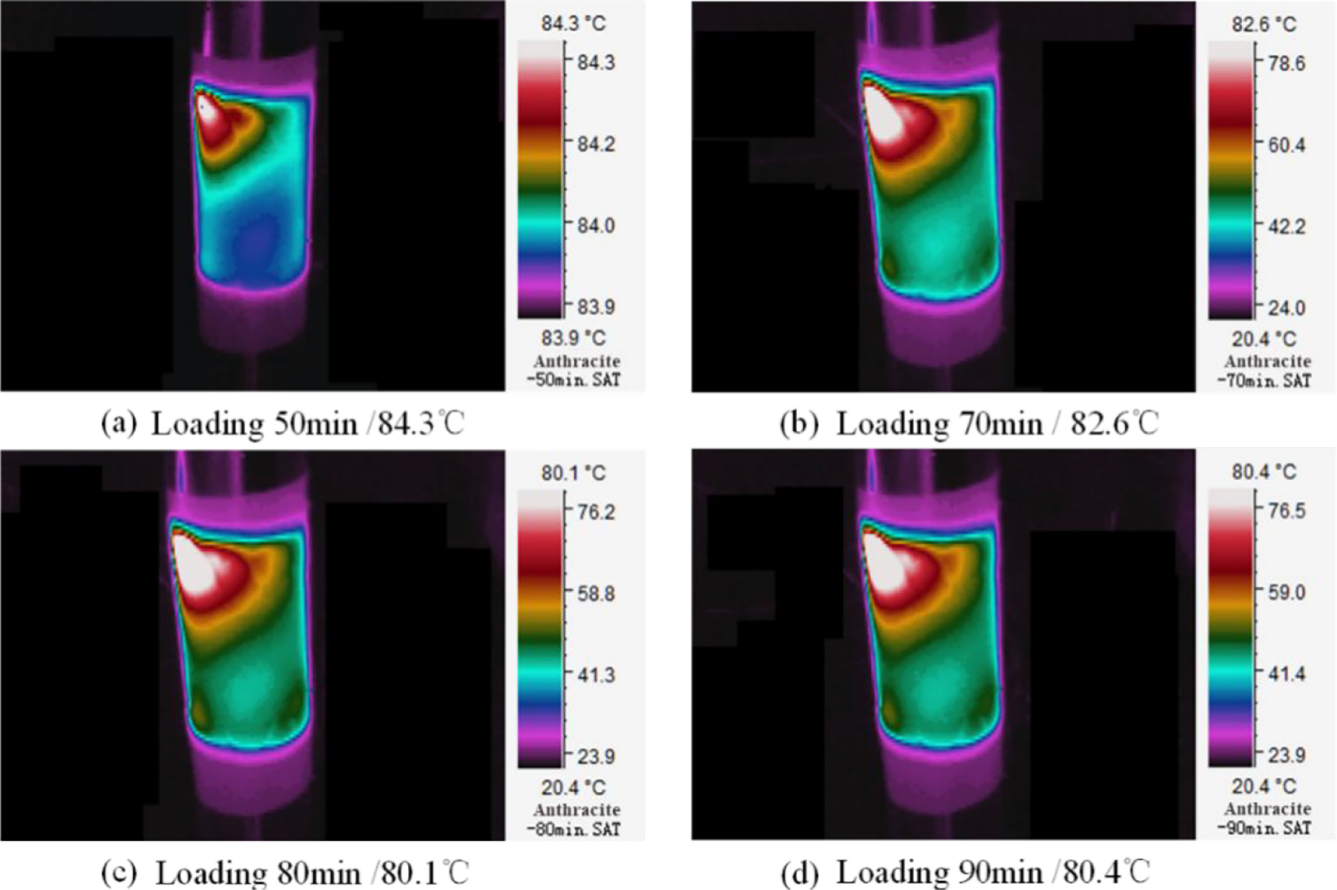
Three-dimensional infrared images of temperature distribution on the surface of anthracite at the slow cooling stage (a to d are different loading times, 50–80 min).

Here, anthracite with the largest temperature change range is taken as the example to accurately characterize and reflect the distribution and continuous change process of temperature on the surface of CEEDCEF and to verify the reliability of numerical simulation. The infrared images of its surface temperature distribution at the stage of slow cooling into stabilization (45–90 min, C–D) are displayed in Figure 10.

It is observed from Figure 10 that the temperature distribution on the surface of the coal sample is heterogeneous. To be specific, high temperature is mainly concentrated near the upper end face of the electrode, showing local accumulation of heat. This is attributed to the unevenly distribution of minerals in the coal sample and the discrepancy in their specific heat capacity. Since the experimental environment is open and the coal sample is not adiabatically treated, the sample is directly exposed to the environment, contacting and exchanging heat with the ambient air by radiation. In this case, if the Joule heat generated by the current passing through the coal sample is below or equal to the heat escaping from the coal to the external environment, the electrical parameters and surface temperature of the coal sample will decrease and tend to stabilize.

At this stage, the change of the highest temperature on the coal sample surface ranges from 84.3 to 80.4 °C, with a temperature change Δ*T* of −3.9 °C and an average temperature rise rate *T̅*_v_ of about −0.08 °C/min. In addition, the surface temperature of the coal sample decreases slowly and finally stabilizes at 80.4 °C, which is consistent with the numerical simulation results.

## 5 Discussion and Analysis

Coal is a unique dielectric material. Its inner free state electrons will transform from haphazard dispersion to oriented arrangement under the action of the electric field, forming a moving current beam.^32^ When the excitation voltage on the coal is below the breakdown voltage, the electric field bears an insignificant effect on the pore structure of the coal even though it has excited the coal for a long time. Meanwhile, the electrons in the coal will attach to the pore wall to generate a field strength between pores. Thus, the electric field energy input from the outside is not released into the pore structure of the coal.

**Figure 11:**
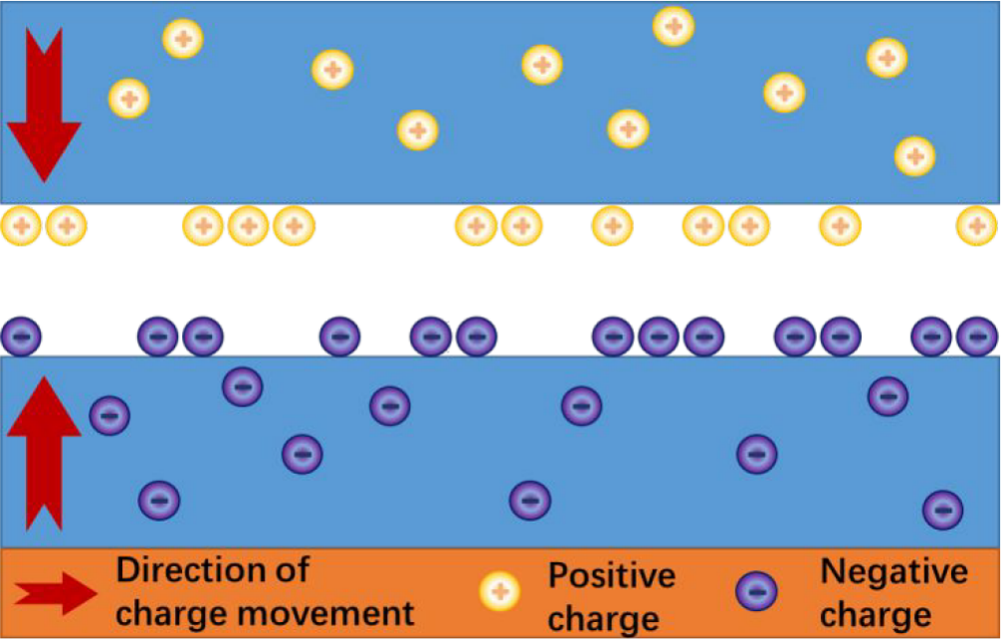
Charge distribution on the wall of pores and fractures at the initial stage of external DC electric field excitation

The charge distribution on the surface of the internal pore fracture of the coal at the initial stage of DC electric field excitation is shown in Figure 11. As can be seen from Figure 11, the energy input from the external electric field accumulates on the wall of pores and fractures inside the coal in the form of electric charge, so that the electric field is formed on the wall. At the initial stage of DC electric field excitation, the charge on the wall of pores and fractures inside the coal sample does not reach saturation. Hence, the coal sample presents a “capacitance effect” when being charged by the electric field. At this time, the current through the coal is low and the Joule heat effect is weak. Macroscopically, the temperature on the surface of the coal rises slowly.

**Figure 12:**
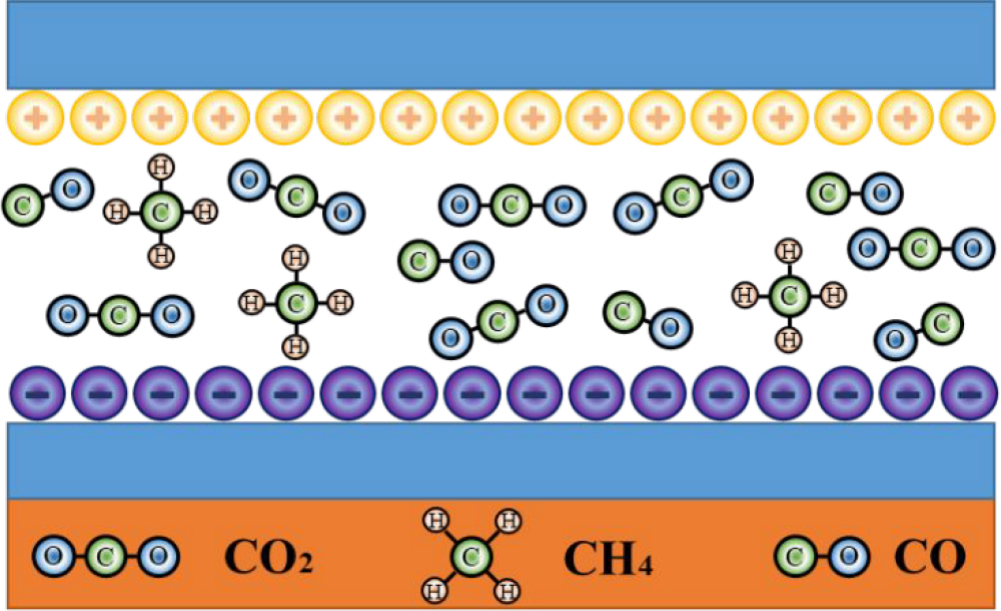
Electric field distribution of the wall of the pores and fractures inside the coal in the state of charge saturation.

The electric field distribution after DC electric field continuously excites the wall of pores and fractures until the charge reaches saturation is displayed in Figure 12. It can be observed that the field strength of the wall of pores and fractures gradually stabilizes after the charge on the wall reaches saturation. The mostly nanoscale pore-fracture spacing inside the coal leads to a strong field strength, which changes the internal surface microstructure of the coal. In particular, the low-bond energy alkane side chains and functional groups on the coal surface are liable to break and fall off to generate new small-molecular gases (such as CO_2_, CH_4_, and CO in Figure 12),^33^ resulting in the change in electrical parameters of the coal sample. The current through the coal sample surges as the equivalent resistance decreases. Meanwhile, the temperature soars as well, due to the Joule effect and the consumption of the dielectric inside the coal.

With the further increase in the time of DC electric field excitation, the internal dielectric of the coal gradually stabilizes and the temperature rise rate changes. When the Joule heat generated by the electric field excitation become lower than or equal to the heat escaping from the coal to the external environment, the surface temperature tends to decrease slowly and stabilizes.

## 6 Conclusions

The main findings of this study are as follows:

(1) The key factors influencing the temperature rise of CEEDCEF are the loading voltage and the loading time. According to the simulation calculation, the temperature of the coal rises notably with the increase in the two key factors.
(2) The simulation results demonstrate that at a loading time of 90 min and a loading voltage of 6 kV, the internal temperature and the maximum surface temperature of the coal reach 94 and 85.5 °C, respectively. The experimental results suggest that the highest temperature on the coal surface ranges from 84.3 to 80.4 °C. The two results are basically consistent in terms of the highest surface temperature of the coal.
(3) Through the comprehensive analysis on the results of numerical simulation and experimental determination, it is found that the energy conversion and temperature rise process of CEEDCEF can be divided into three stages, i.e., slow warming, fast warming, and slow cooling into stabilization.
(4) From the microscopic perspective, at the early stage of DC electric field excitation, the electrons inside the coal continuously accumulate on the wall of pores and fractures, so that the charge reaches saturation. At this time, the field strength between the wall prompts the free radicals in the coal molecular structure to shake off their original bondage and jump toward the direction of the electric field, resulting in an increased current that heats the coal rapidly. Under continuous excitation of the DC electric field, the electrical parameters of the coal tend to be stable, and the surface temperature of the coal tends to stabilize.
